# Higher Fertilizer Inputs Increase Fitness Traits of Brown Planthopper in Rice

**DOI:** 10.1038/s41598-017-05023-7

**Published:** 2017-07-05

**Authors:** M. M. Rashid, N. Ahmed, M. Jahan, K. S. Islam, C. Nansen, J. L. Willers, M. P. Ali

**Affiliations:** 1 Plant Physiology Division, Bangladesh Rice Research Institute (BRRI), Gazipur, 1701 Bangladesh; 2 Entomology Division, Bangladesh Rice Research Institute (BRRI), Gazipur, 1701 Bangladesh; 3 Department of Entomology, Bangladesh Agricultural University (BAU), Mymensingh, 2202 Bangladesh; 4 Department of Entomology and Nematology, UC Davis Briggs Hall, Room 367, Davis, CA USA; 5 State Key Laboratory Breeding Base for Zhejiang Sustainable Pest and Disease Control, Zhejiang Academy of Agricultural Sciences, 198 Shiqiao Road, Hangzhou, 310021 China; 6 United States Department of Agriculture, Agricultural Research Service, Southern Insect Management Research Unit, 141 Experiment Station Road, P.O. Box 346, Stoneville, MS 38776 USA

## Abstract

Rice (*Oryza sativa* L.) is the primary staple food source for more than half of the world’s population. In many developing countries, increased use of fertilizers is a response to increase demand for rice. In this study, we investigated the effects of three principal fertilizer components (nitrogen, phosphorus and potassium) on the development of potted rice plants and their effects on fitness traits of the brown planthopper (BPH) [*Nilaparvata lugens* (Stål) (Homoptera: Delphacidae)], which is a major pest of rice in Bangladesh and elsewhere. Compared to low fertilizer inputs, high fertilizer treatments induced plant growth but also favored BPH development. The BPH had higher survival, developed faster, and the intrinsic rate of natural increase (*r*_*m*_) was higher on well-fertilized than under-fertilized plants. Among the fertilizer inputs, nitrogen had the strongest effect on the fitness traits of BPH. Furthermore, both the “Plant vigor hypothesis” and the “Plant stress hypothesis” were supported by the results, the former hypothesis more so than the latter. These hypotheses suggest that the most suitable/attractive hosts for insect herbivores are the most vigorous plants. Our findings emphasized that an exclusive focus on yield increases through only enhanced crop fertilization may have unforeseen, indirect, effects on crop susceptibility to pests, such as BPH.

## Introduction

Rice is the primary staple food source for over half the world’s population. The crop is cultivated in at least 114 (mostly developing) countries and is the primary source of income and employment for more than 100 million households in Asia and Africa^1^. In recent years, especially in developing countries, rice production has not matched the food demands of an increasing population^2^. To meet this growing demand, rice production has to be raised by at least 70% over the next three decades^3^. The land area devoted to rice cultivation is limited and production cannot be increased by more acreage^3^, so additional, applied research is needed to find other ways of increasing productivity. With limited land resources and increased demand for enhanced production attention is turning towards intensification through higher fertilizer inputs, which is predicted to result in higher yields.

Despite a sound logic base supporting increased fertilizer inputs in some rice cropping systems, possible indirect adverse effects of increased fertilizer inputs were highlighted by Heong^4^. In the last decade, planthopper (Homoptera: Delphacidae) outbreaks in rice fields have intensified across Asia resulting in heavy yield losses^5, 6^. Over the past decade yield losses substantially increased due to a widespread outbreak of the brown plant hopper (BPH) [(*Nilaparvata lugens* Stål)]^5, 7^. For example, the Central Plains of Thailand suffered from persistent planthopper outbreaks for 10 consecutive growing seasons from 2008 to 2012 and caused losses worth $52 million or equivalent to about 173,000 tons in 2010^6^. The same pest was responsible for losses of around 1 million tons in Vietnam in 2007, and resulted in a government freeze on rice exports^6^.

Relationships among fertilizer applications and insect pest outbreaks are widely described in the scientific literature, especially in response to nitrogen fertilization^8–13^. Specifically regarding rice-based cropping systems, there is a considerable body of research highlighting the indirect effects of fertilizer applications on crop susceptibility to pests. As an example, BPH prefer to feed and oviposit on rice plants supplied with nitrogen^14–16^. BPH reared on plants with high nitrogen content had high feeding rates and honeydew excretion^14, 17^, less probing behavior^15, 17^, higher survival rates and population build-ups^18–20^, higher fecundity and oöcyte production^16, 19^, and higher risk of economically important BPH outbreaks^21–23^. Nitrogen fertilization has also significantly increased the populations of white-backed planthoppers (*Sogatella furcifera* Horváth)^24, 25^, green leafhoppers (*Nephotettix virescens* Distant)^24, 25^, and small brown planthoppers (*Laodelphax striatellus* Fallén)^25^. Finally, Pandey^26^ reported higher incidence of rice leafrollers (*Cnaphalocrocis medinalis* Guénee) damage at higher levels of nitrogen fertilization.

Phosphorus fertilization has been reported to markedly increase population growth of BPH^27^. Phosphorus alone or combinations with nitrogen and nitrogen-phosphorus-potassium treatments are reported to support moderate leafhopper populations^28^. Treatments with phosphorus alone and phosphorus in combination with nitrogen also increased populations of ear head bugs (*Leptocorisa oratorius* F.) and associated grain damage^2^. It has been suggested that phosphorus tends to increase abundance of yellow stem borers (*Scirpophaga incertulas* Walker) in rice, but to a lesser degree than nitrogen^2^. High fertilization levels of both nitrogen and phosphorus caused higher levels of damage by blue beetles (*Leptispa pygmaea* Baly) in rice crops^28^.

Regarding potassium fertilization, there are reports of negative associations between application rates and prevalence of insect pests in rice^29^. As an example, Kulagold *et al*.^28^ reported that higher potassium fertilization of rice plants led to reduced densities of green leafhoppers, yellow stem borers, blue beetles, rice leafrollers and ear head bugs. The rate of rice stem borer infestation was greatest when there was no supply of potassium, but decreased in response to increased potassium concentration in rice plants^30^.

Silicon (Si) content in rice is reported several folds higher than N, P, and K^31, 32^, also promoting a beneficial effect in rice. Recently Guntzer *et al*.^33^ reported that Si increases resistance against insect pests, pathogens and abiotic stresses including salinity, drought and storms. In this research, N * P * K interactions in a factorial experiments upon Si content of rice is one of the measured attributes.

Ample evidence supports a general hypothesis that excessive (or unbalanced) crop fertilization regimes affects both risk of infestation and severity of economically insect-induced crop losses. However, there are few studies where the combined effects of nitrogen, phosphorous, and potassium fertilizers are studied in detail regarding BPH infestations and its life history characteristics on rice, and also as collectively altered by varying Si content of rice plant tissues. Our previous study showed that biochemical constituents of BPH varied with nutrient levels at different growth stages, and changes in relative water content (RWC) of rice plants^34^. Moreover, concentrations of N and P were found much higher in the BPH body than in its host rice plants, and this elemental mismatch is an inherent constraint on meeting nutritional requirements of BPH. In this study, rice plants were grown in pots under factorial combinations of fertilizer regimes (nitrogen, phosphorous and potassium) and subsequently assessed as host plants for BPH. The following specific objectives were to explore direct effects of NPK fertilizer regimes on physiological characteristics of rice plants, and indirect effects of these regimes on fitness traits of BPH.

## Results

### Principal Component Analysis - Physiological characteristics of rice plants

**Figure 1:**
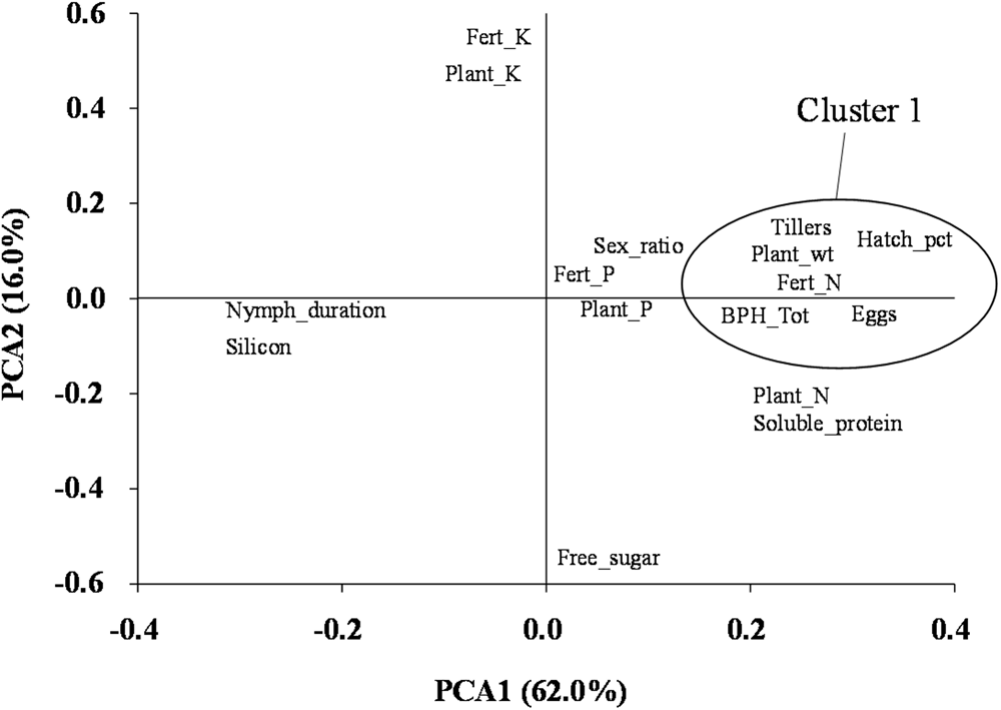
Physiological characteristics of rice plants and their effects on brown planthopper (BPH) fitness traits.

The principal component analysis of fertilizer regimes, nutritional elements of rice plants, and BPH fitness trait responses showed that 78% of the total variance could be explained by the two principal axes, PCA1 and PCA2 (Fig. 1). To improve visualization, four variables (plant height, BPH nymph survival, BPH adult longevity, and total BPH fresh weight) were not included in Fig. 1, but they were located within the space denoted “cluster 1”. Due to the high level of variance explained in the two-dimensional space defined by the principal axes, we could make fairly strong inferences about the relative associations of the explanatory variables to show that: 1) there were very close associations between fertilizer regimes and the corresponding content of the same macro elements in rice plants; 2) all agronomic rice plant traits (except plant content of potassium and silicon) were positively associated with nitrogen fertilization (and nitrogen content of rice plants); 3) nitrogen fertilization was strongly associated with PCA1 explaining most of the variance in the data set; 4) the PCA2 axes was clearly associated with a negative relationship between free soluble sugar and potassium in rice plants.

### Effect of fertilizer inputs on the fitness traits of BPH

Nitrogen fertilization significantly increased BPH survival from egg to nymph or to adult and all other fitness traits (except nymphal duration) were positively correlated, which indicated these traits improved significantly with the increase of nitrogen inputs (Fig. 1). Fitness responses by BPH to phosphorus showed the same trend as nitrogen, but the level of association was more modest. There was a negative association between potassium and soluble sugar (at opposite ends of the second principal axis, PCA2), and these two plant nutrition traits were orthogonal to the BPH fitness traits located along the principal axis, PCA1. This suggested that potassium and soluble sugar had only a minor influence on BPH fitness traits. Very interestingly, BPH nymphal development time was positively associated with silicon plant content and negatively correlated with all other principal BPH fitness traits.

### Regression modeling of BPH fitness traits

**Figure 2:**
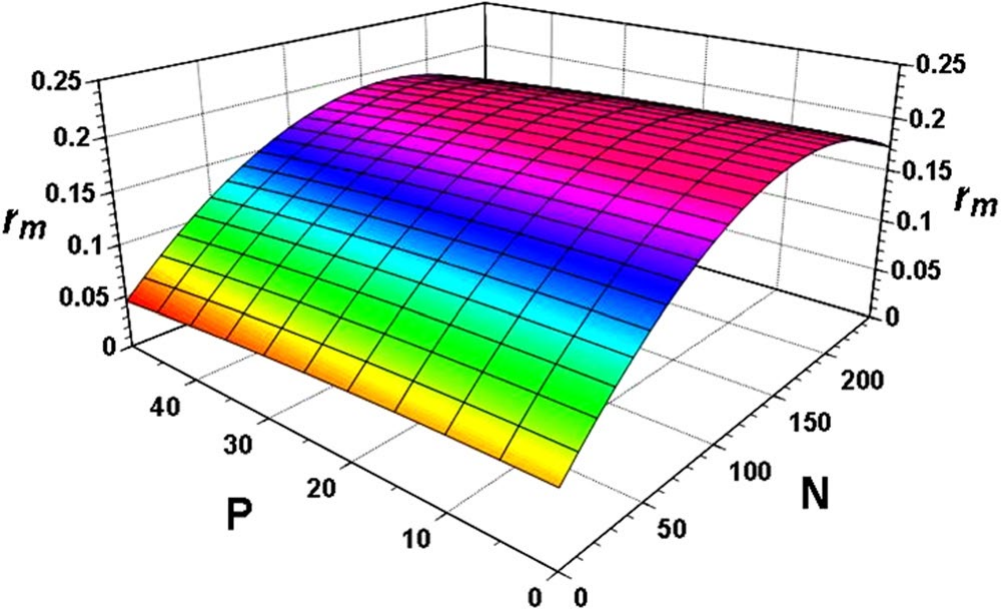
Effect of N and P on the increasing rate of natural increase (*r*_*m*_) of brown planthopper (BPH).

**Figure 3:**
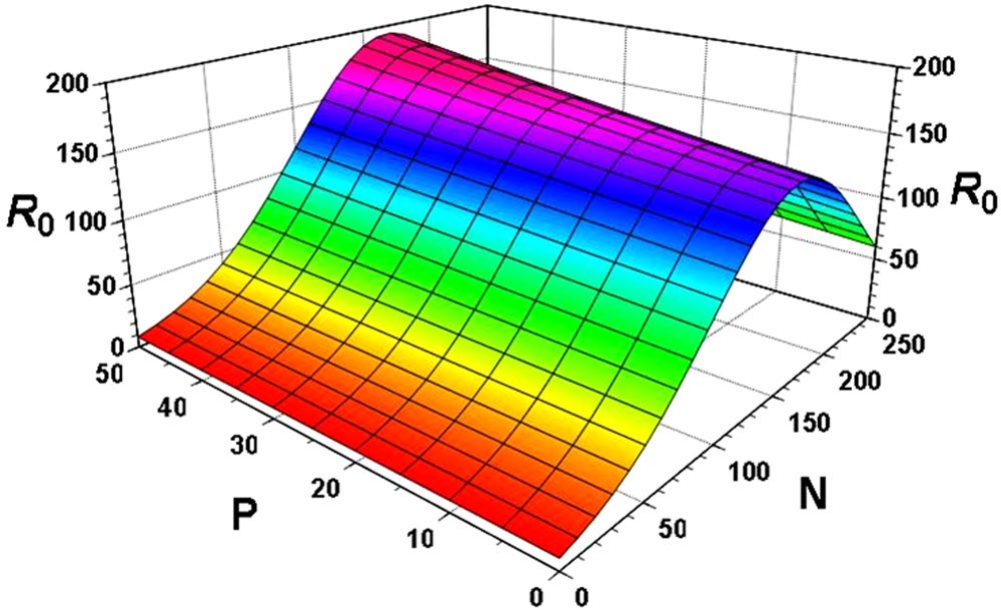
Effect of N and P on the reproduction rate (*R*_*0*_) of brown planthopper (BPH).

The intrinsic rate of natural increase (*r*_m_), the finite rate of increase (*λ*), and net reproductive rate (*R*_0_) significantly increased in response to nitrogen fertilization. However, phosphorus and potassium fertilizers individually did not have a direct, significant effect on these fitness traits (p > 0.05); thus, all of our linear modelling analyses supported the findings found by the principal component analysis. However, phosphorus and potassium were applied with nitrogen, the intrinsic rate of natural increase (*r*_m_), the finite rate of increase (*λ*) and reproduction rate (*R*_0_) increased most significantly with only the higher nitrogen regime (p < 0.05). The full model with all fertilizers explained 97.5% of the total variation of the intrinsic rate of natural increase (*r*_*m*_). Overall, nitrogen had higher predictive power than phosphorus, and potassium had no significant effect on demographic parameters of BPH, we next emphasized nitrogen and phosphorus in further analyses. Using the STEPWISE option in GLMSELECT, analyses reduced the full model to *r*_*m*_ = 0.0639 + 0.00056 *P* + 0.00017 *N* − 0.0000076*P* ^2^ − 0.0000051*N* ^2^, which continued to explain 97.5% of the total variation (*F*_4,103_ = 995.3; *P* < 0.0001). In the case of BPH net reproductive rate (using a natural log transformation), ln *R*_0_ = 2.1105 + 0.03618*N* − 0.000113*N* ^2^ + 0.0000315 *P* * *N*, and explained 98.6% of the total variation in data (*F*_3,104_ = 2454.9; *P* < 0.0001) (The predicted values of *R*_0_ were obtained by taking the antilog). Figures 2 and 3 showed the predicted combined effects of nitrogen and phosphorus on *r*_*m*_ and *R*_*0*_ of BPH. The mean length of a generation (*t*_G_) and population doubling (DT) time decreased significantly with increasing N inputs (p < 0.001).

### MANOVA Analyses - Physiological characteristics of rice plants

MANOVA analyses indicated a significant multivariate effect among treatment combinations within levels of Nitrogen. For N_0_, Wilks’ lambda = 0.0004, *F*(40, 63.819) = 8.06; *p* < 0.0001, for N_100_, Wilks’ lambda <0.0001, *F*(40, 63.819) = 13.13; *p* < 0.0001, and for N_200_, Wilks’ lambda <0.0001, *F*(40, 63.819) = 12.32; *p* < 0.0001. These results indicate that there are differences between the groups over the levels of the N factor for assayed physiological characteristics. The univariate ANOVAs of these physiological characteristics were next examined.

**Figure 4:**
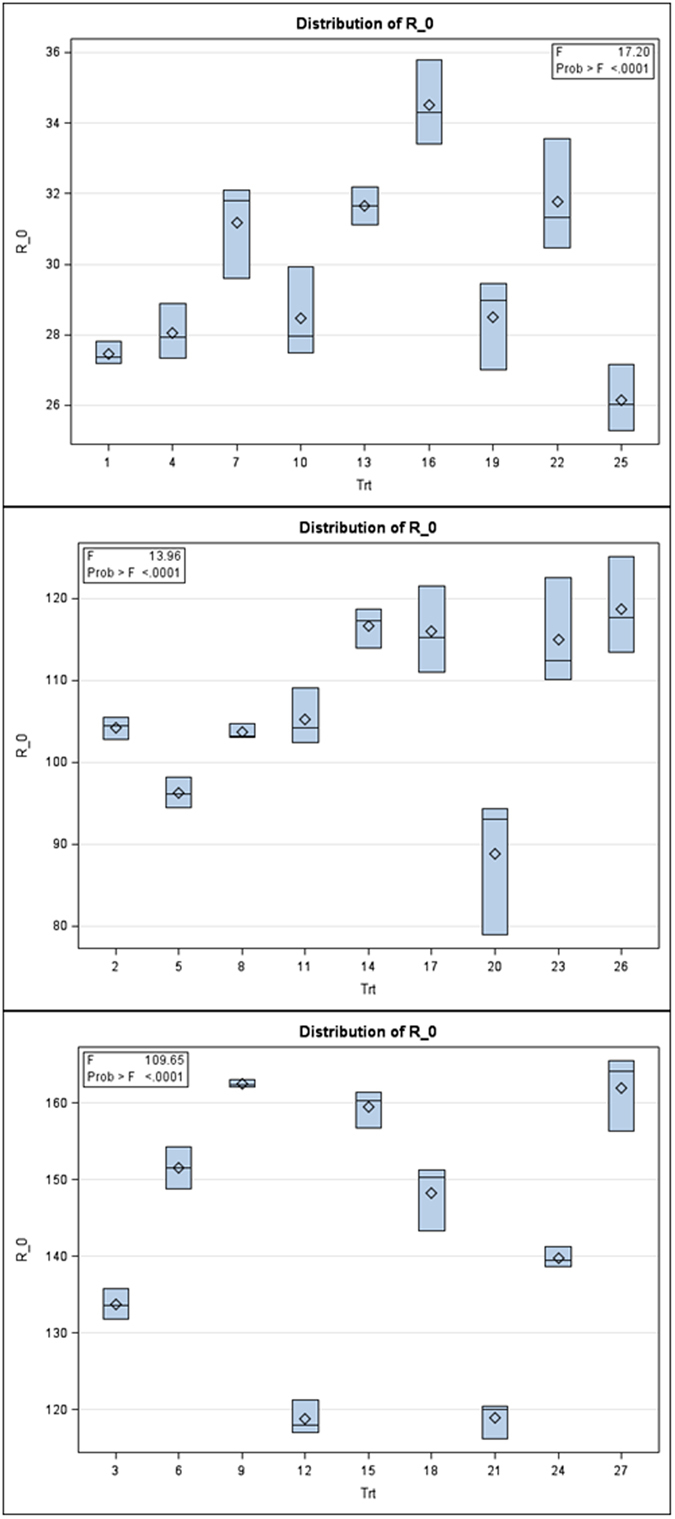
Univariate Box plot results following multivariate MANOVA for the fitness trait *r*_*m*_ (labeled as R_m in the figures) over the 3 levels of the N factor. Note the subtle pattern for the group means (in sets of 3 moving by eye from left to right) in each panel and among panels.

**Figure 5:**
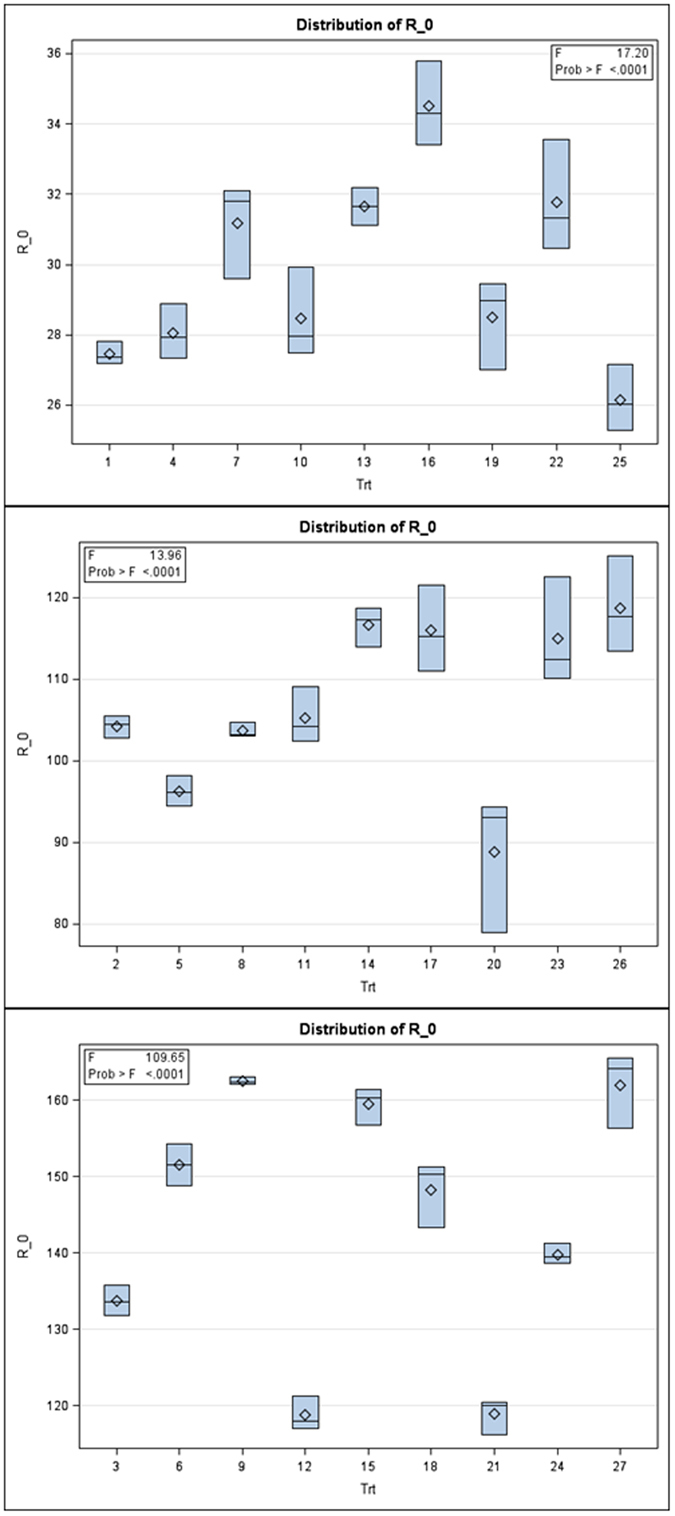
Univariate Box plot results following multivariate MANOVA for the fitness trait *R*_0_ (labeled as R_0 in the figures) over the 3 levels of the N factor. Note the subtle pattern for the group means (in sets of 3 moving by eye from left to right) in each panel and among panels.

Interestingly, subtle patterns were found among fitness traits with the physiological characteristics of the rice plants using MANOVA. Granting the dominant results discussed in the previous paragraphs, it follows that subtle patterns with *r*_m_ and *R*_0_ are also indicated using MANOVA where the levels of nitrogen are the BY variable in general linear model analyses (SAS PROC GLM). These analyses indicated a significant multivariate effect for *r*_m_ and *R*_0_ among the nine treatment combinations within each of the three levels of the N factor (Figs 4 and 5). For *r*_m_ and *R*_0_, at the lowest level of nitrogen, Wilks’ lambda = 0.0084, *F*(16, 34) = 21.02; *p* < 0.0001, for the middle nitrogen rate, Wilks’ lambda = 0.0416, *F*(16, 34) = 8.30; *p* < 0.0001, and for the highest nitrogen rate, Wilks’ lambda = 0.0051, *F*(16, 34) = 12.32; *p* < 0.0001, which indicate there are differences between groups over the levels of the N factor for these assayed physiological characteristics. The univariate ANOVAs were again examined, with another emphases upon the subtle patterns in the Box plot outputs for both fitness traits of BPH. These Box plots succinctly show why the multivariate MANOVA of fitness traits results were highly significant at all levels of the N factor (Figs 4 and 5).

**Figure 6:**
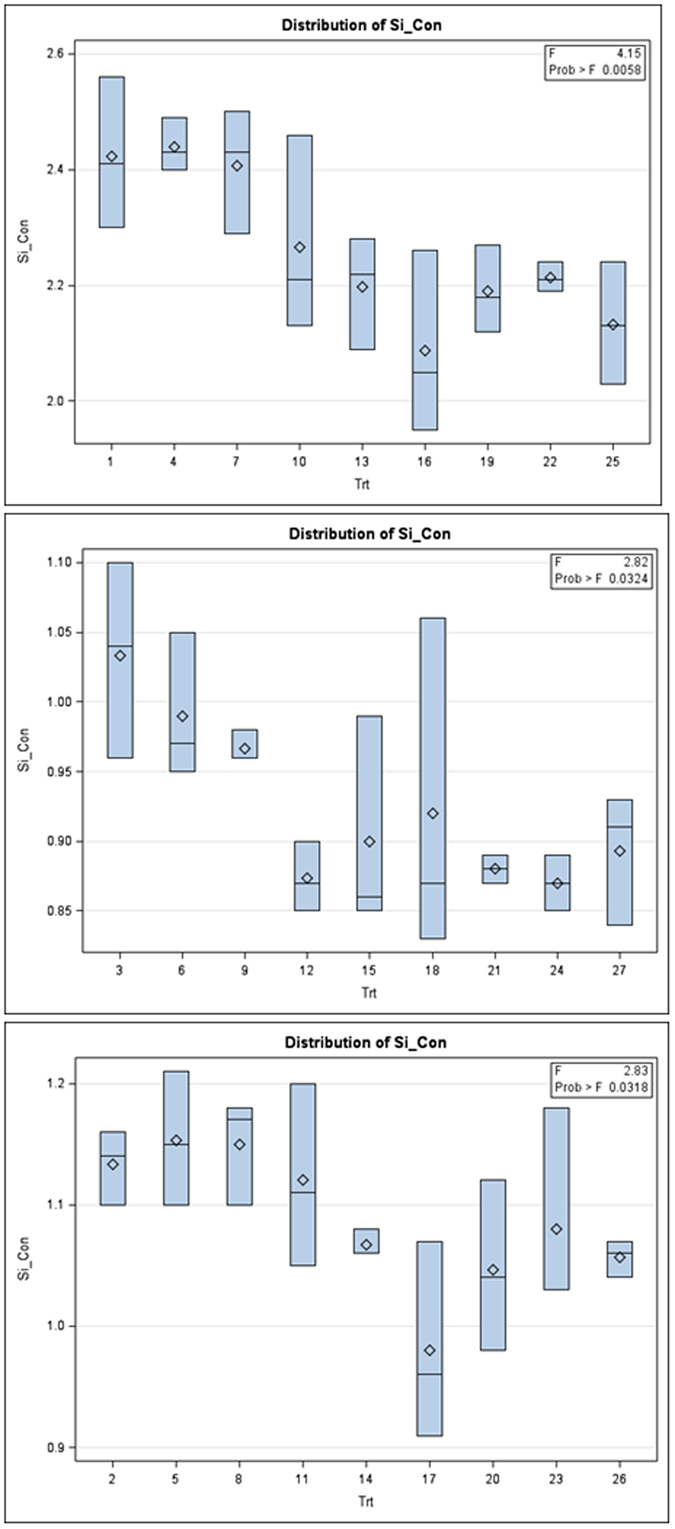
Univariate Box plot results following multivariate MANOVA for the silicon (Si) content plant characteristic over the 3 levels of the N factor. Compare the subtle differences in eye fit lines (in sets of 3 groups, moving left to right) within each sub-panel and among the sub-panels.

These findings indicate that NPK and Si effects are involved in two-way or higher order interactions, which influence BPH fitness traits to different degrees. For instance, if just the Box plots for the characteristic, silicon content (Fig. 6), are examined it is readily seen that these plots differ in shape amongst the N levels, and are found to do so more than the Box plots for other characteristics (hence, for reasons of brevity, Box plot results for other measured characteristics are not presented). While the F-value is significant, silicon content has the smallest set of F-values (see insert, upper right of Fig. 6) compared to the other four physiological characteristics (Table 1).

**Table 1:** One-way MANOVA results summary for five rice plant characteristics.

Characteristic	*df*	*Type III SS*	*Mean Square*	*F Value*	*Pr* > *F*
**N**_**0**_					
N Content	8	0.07300000	0.00912500	12.26	<0.0001
P Content	8	0.03578519	0.00447315	33.55	<0.0001
K Content	8	1.55438519	0.19429815	31.17	<0.0001
Sol. Protein	8	1.94087407	0.24260926	27.59	<0.0001
Si Content	8	0.41334074	0.05166759	4.15	0.0058
**N**_**100**_					
N Content	8	1.25485185	0.15685648	55.65	<0.0001
P Content	8	0.05102963	0.00637870	44.16	<0.0001
K Content	8	1.63673333	0.20459167	64.76	<0.0001
Sol. Protein	8	5.13742963	0.64217870	129.78	<0.0001
Si Content	8	0.07818519	0.00977315	2.83	0.0318
**N**_**200**_					
N Content	8	1.08278519	0.13534815	39.98	<0.0001
P Content	8	0.05214074	0.00651759	70.39	<0.0001
K Content	8	1.59013333	0.19876667	29.10	<0.0001
Sol. Protein	8	3.67005185	0.45875648	90.41	<0.0001
Si Content	8	0.08120741	0.01015093	2.82	0.0324

Since there are many ways to examine the MANOVA output, we were led to examine perspectives of the data at the juncture of many subtle effects. We suggest that our usage of stylized, multi-dimensional plots of N, P, K, N_Con, P_Con, K_Con, Si_Con, R_m and R_0 relationships aid interpretation of the findings than otherwise by the more traditional statistical tools. The usage of symbol color, symbol shape, symbol size, and a surface grid comprise the various style elements that convey relationships among the variates.

#### Stylized Multi-dimensional Plots of Subtle effects

**Figure 7:**
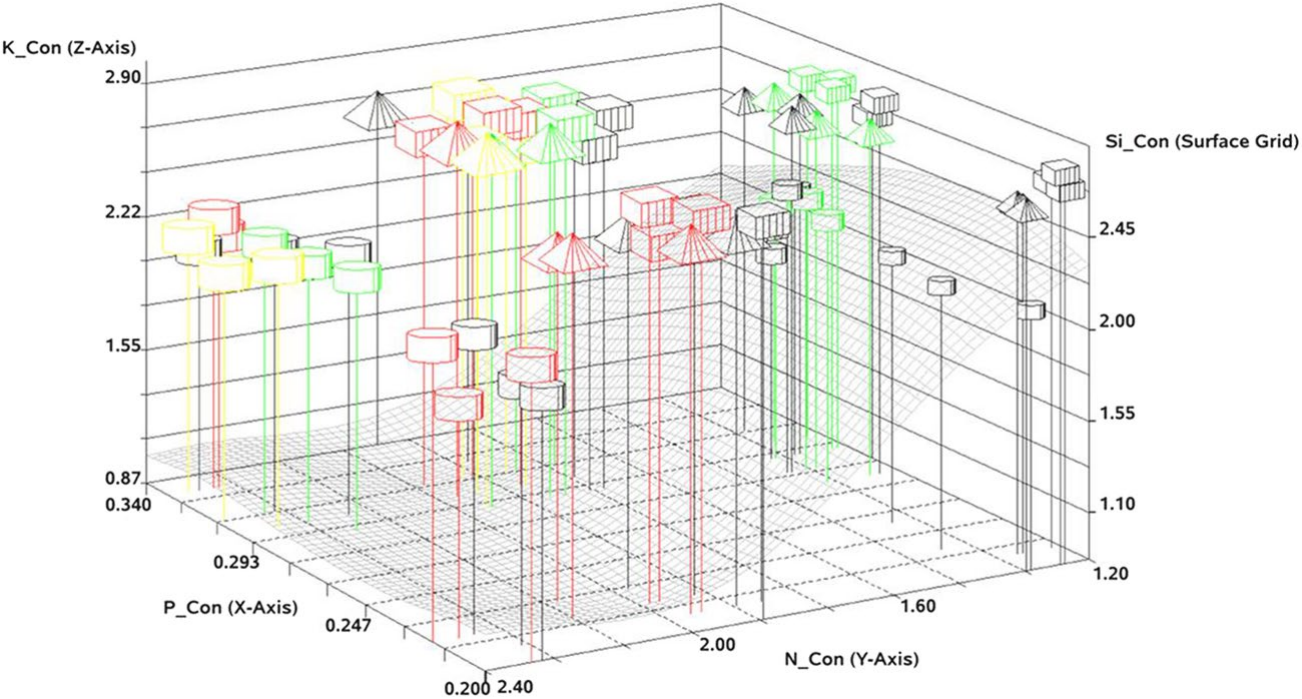
Relationship among measured rice plant attributes and brown planthopper (BPH) fitness traits for intrinsic rate of increase (*r*_*m*_).

**Figure 8:**
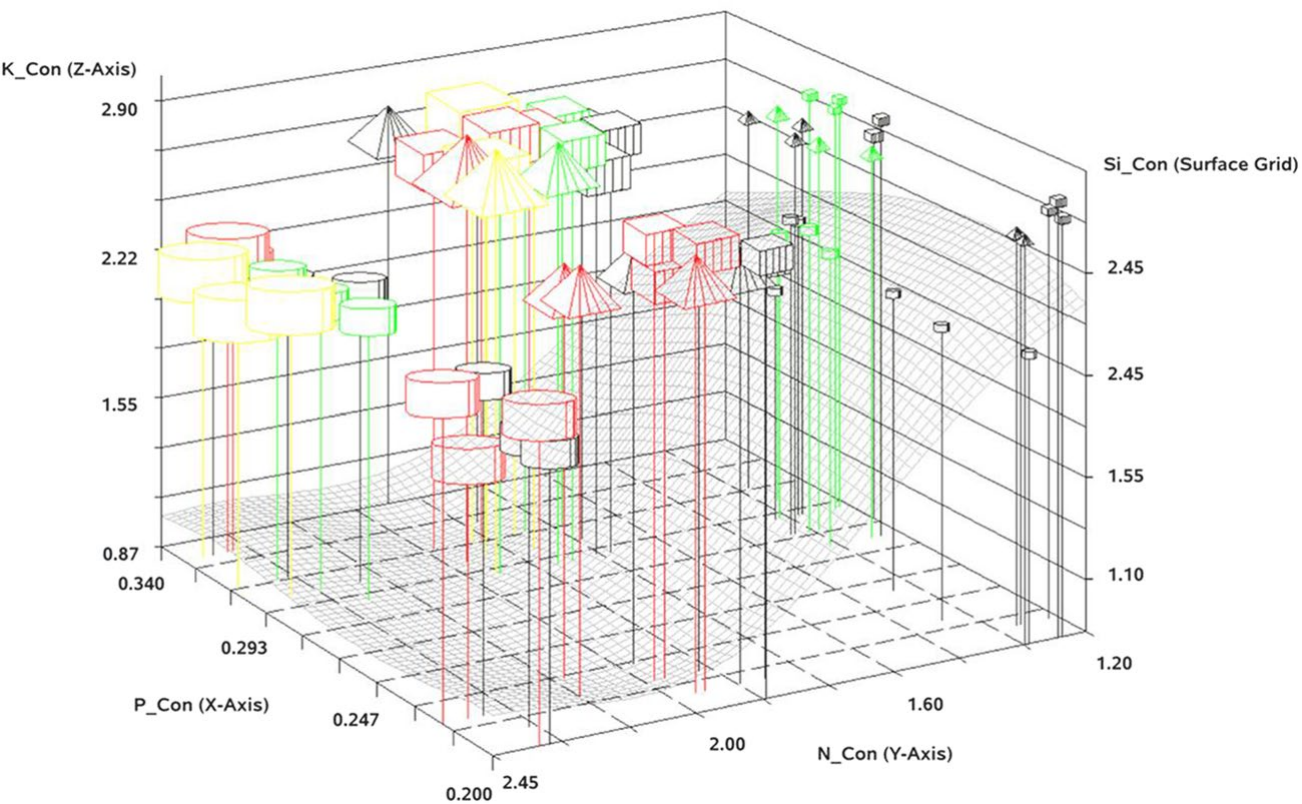
Relationship among measured rice plant attributes and brown planthopper (BPH) fitness traits for net reproductive rate (*R*_0_).

Two colorful multi-dimensional plots (Figs 7 and 8) that convey information in more than just 3-dimensions employed symbol shape, symbol color, and symbol size to convey more relationships among the data attributes than just tables of means and levels of significance expressed as p-values. Here, the X-axis plots the P content, the Y-axis plots the N content and the Z_1_-axis plots the K content of the rice plant observations as associated with the factor levels of the experiment (Tables 2 and 3). For example, if the symbol is a cylinder, the amount of the K fertilizer applied to the soil was 0, if the symbol is a pyramid, the amount applied was 60, and if the symbol is a cube, the amount of K fertilizer applied was 120. The sizes of these three symbols are driven by either *r*_m_/0.05, or by *R*_0_/30. Various colors assigned to these symbols shapes of the various sizes are determined by RGB values, where the minimum and maximum levels of the N and P fertilizers are found by the percent of range transform^35^.

1 $$Gray\,Scale\,Valu{e}_{(R=NorG=P)}=\frac{(Fertilizer\,Level-Minimum)}{(Maximum-Minimum)}\,\ast \,{255}$$

**Figure 9:**
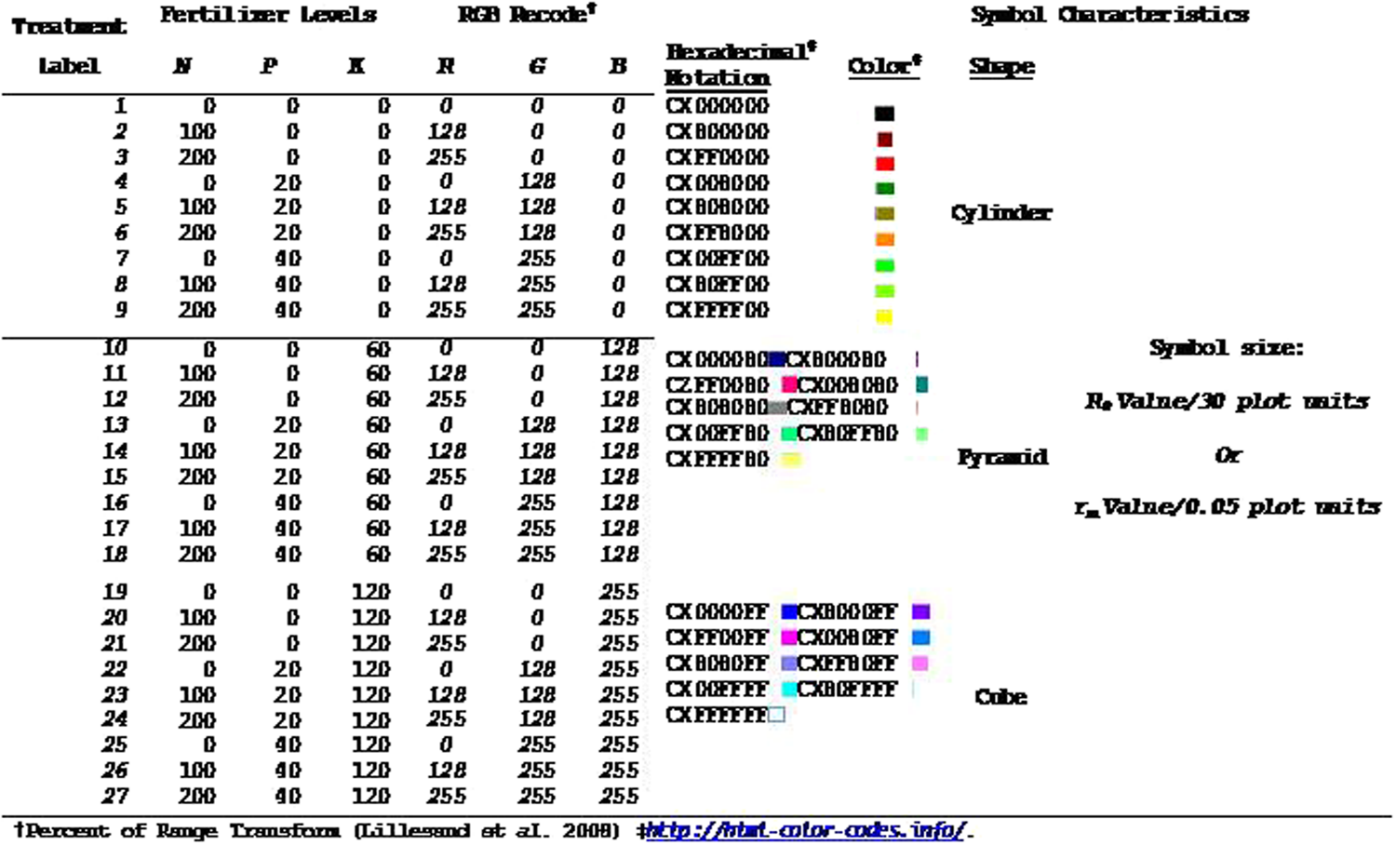
Legend for *3*-*D* graphics and symbology characteristics for fertilizers, life history traits of BPH and bioassay results.

Since K was used to define the symbol shape, the B (blue) value of this RGB color code was 0 for all levels of K. The resultant table was converted into hexadecimal color codes for plotting by SAS/GRAPH PROC G3D using the SCATTER option, and these results produce the legend table (Fig. 9) for the stylized multi-dimensional plots.

**Table 2:** Summary statistics from one-way MANOVA of treatment groups for the level of the N factor = 0 units, which are used for multi-dimensional plotting with BPH fitness criteria.

Trt ID	N	N_0_									
		N_Con		P_Con		K_Con		Sol_Protn		Si_Con	
		Mean	Std Dev	Mean	Std Dev	Mean	Std Dev	Mean	Std Dev	Mean	Std Dev
1	3	1.36333333	0.03785939	0.22000000	0.02000000	2.17000000	0.03000000	2.53000000	0.07549834	2.42333333	0.13051181
4	3	1.35000000	0.03605551	0.28666667	0.00577350	2.15000000	0.20420578	2.51333333	0.08621678	2.44000000	0.04582576
7	3	1.34666667	0.00577350	0.28000000	0.01000000	2.20666667	0.04041452	2.54333333	0.05686241	2.40666667	0.10692677
10	3	1.30000000	0.02645751	0.20666667	0.00577350	2.61666667	0.03055050	1.92666667	0.05507571	2.26666667	0.17214335
13	3	1.32666667	0.03785939	0.29333333	0.01527525	2.65000000	0.04358899	1.93666667	0.06806859	2.19666667	0.09712535
16	3	1.29333333	0.03055050	0.28333333	0.01527525	2.64666667	0.02081666	2.01333333	0.06658328	2.08666667	0.15821926
19	3	1.24333333	0.02081666	0.20333333	0.00577350	2.72666667	0.05686241	1.94666667	0.14153916	2.19000000	0.07549834
22	3	1.22000000	0.02000000	0.28000000	0.01000000	2.70333333	0.07023769	1.99000000	0.14177447	2.21333333	0.02516611
25	3	1.22666667	0.00577350	0.29333333	0.00577350	2.72666667	0.02081666	1.96333333	0.10263203	2.13333333	0.10503968

**Table 3:** Summary statistics from one-way MANOVA of treatment groups for the level of the N factor = 100 units, which are used for multi-dimensional plotting with BPH fitness criteria.

Trt ID	N	N_100_									
		N_Con		P_Con		K_Con		Sol_Protn		Si_Con	
		Mean	Std Dev	Mean	Std Dev	Mean	Std Dev	Mean	Std Dev	Mean	Std Dev
2	3	2.29666667	0.02081666	0.21000000	0.01000000	2.17666667	0.10692677	4.15666667	0.03511885	1.13333333	0.03055050
5	3	2.26666667	0.08082904	0.31333333	0.01527525	2.15000000	0.05196152	4.21333333	0.15502688	1.15333333	0.05507571
8	3	2.25666667	0.04509250	0.30000000	0.01000000	2.17000000	0.04582576	4.22000000	0.09539392	1.15000000	0.04358899
11	3	1.93666667	0.06658328	0.22000000	0.01000000	2.64333333	0.03214550	3.28666667	0.06658328	1.12000000	0.07549834
14	3	1.88666667	0.04509250	0.32000000	0.02000000	2.62000000	0.02000000	3.28666667	0.02516611	1.06666667	0.01154701
17	3	1.85000000	0.07937254	0.29666667	0.01154701	2.66333333	0.02081666	3.27666667	0.03055050	0.98000000	0.08185353
20	3	1.85333333	0.04041452	0.21000000	0.01000000	2.73000000	0.04582576	3.26666667	0.02516611	1.04666667	0.07023769
23	3	1.72333333	0.01527525	0.29333333	0.00577350	2.70000000	0.07211103	3.26000000	0.05000000	1.08000000	0.08660254
26	3	1.75666667	0.04163332	0.30000000	0.01000000	2.73666667	0.05507571	3.25666667	0.03214550	1.05666667	0.01527525

Using the XYZ axes defined above (and in Figs 7 and 8) a surface grid of the Si content of the rice plants as related to the assay values for N, P, and K was derived by SAS/GRAPH PROC G3GRID, using the SPLINE option and a SMOOTH option value of 0.125, interposed by GIS processing into the plots. The products produced by SAS/GRAPH for the symbols and surface were rasterized in the BMP image format and displayed in ARCGIS 10.2.1 software (ESRI, Redlands, CA). Since the data of these raster layers for the Z1 and Z2 axes was the same range, the coordinate space of the pixels for the XYZ_1_ and XYZ_2_ coordinate space pixels could be interposed into a common scaling within a third, and final, XYZ_*i*_ coordinate space (*i* = 1 for K and 2 for Si). Other GIS tools were used to heads-up digitize the axis labels and values, as well as to reduce the transparency of the Si surface grid to a value of 70% to provide less busy figures. The symbols of each replicate of fertilizer factorial combinations are plotted as just described. At a glance, effects of N and Si as levels of P and K vary on BPH fitness parameters can be related to one another in a multi-dimensional space. Readers are invited to develop an interpretation using legend information presented in Fig. 9 (ours is developed further in the Discussion section).

## Discussion

BPH is one of the most important insect pests of rice in Asia^36, 37^, and it has been an extremely severe pest of rice for decades^5, 6^. However, BPH was a relatively minor rice pest prior to the advent of high-input rice farming which became major after the development and widespread adoption of new high yielding varieties that required increased inputs of fertilizers and pesticides. These practices altered the crop microclimate and accentuated the pest problem such as shifting of the BPH from a minor to a major insect pest^38^.

Our data demonstrate that variation of fertilizer inputs to rice plants significantly affected fitness traits of BPH. Firstly, higher nitrogen input increased the survival rate from egg to nymph or adult stages. Secondly, nitrogen excess increased fresh body weight and enhanced the development of BPH. Thus, higher nitrogen input increased the all fitness traits of BPH. Phosphorus and potassium fertilizers had no significant effect BPH. But if phosphorus is supplied with higher nitrogen input (200 kg/ha), phosphorus significantly influenced several fitness traits including fecundity (eggs/female), fresh body weight and total number of adult BPH developed from one pair of BPH (Table 1). Potassium has no significant effect on any fitness traits of BPH either alone or combined with higher nitrogen input (Fig. 1, Tables 2 and 3), but may as shown by MANOVA (Figs 4, 5 and 6) and stylized 3D plots (Figs 7 and 8), exhibit subtle effects that merit further experiments. These results corroborate the first aim of this study by making it clear that altered fertilizer inputs to rice plants can trigger a bottom-up effect on the fitness traits of BPH. Thus, three questions remain to be answered. What are the physiological changes in the host plants that could explain the effects on the fitness traits of BPH? Do our findings support or refute the “Plant vigor hypothesis” which suggests that herbivorous insects prefer and perform better on rapidly-growing plants^39^? Do our findings support or refute the “Plant stress hypothesis”^39^ and what can learn from this study to better manage rice production by fertilizer by pest relationships?

Different fertilizer treatment combinations influenced rice plant physiology and in turn affected the fitness traits of BPH. When higher doses of nitrogen fertilizer were applied, plants accumulated higher amounts of nitrogen and soluble protein content in their plant tissue, which ultimately influenced herbivore growth and development (Fig. 1). Egg hatchability also increased with nitrogen content, resulting in more BPH produced with higher dry body weights. Between generations, BPH reared on plants receiving 200 kg nitrogen ha^−1^ produced three times more eggs than those reared on plants with no nitrogen applied. Heavy applications of nitrogenous fertilizer may not affect insect biology directly, but they bring about changes in host-plant morphology, biochemistry, and physiology, which can improve nutritional conditions for herbivores^40, 41^. Insects need to transform the inorganic nitrogen forms present in plant tissue and/or utilize directly plant-derived amino acids (i.e., aspartic and glutamic acids^42^) to synthesize structural proteins and enzymes^43^ or nitrogen enriched plants have a lower accumulation of plant allelochemicals which can be less toxic to herbivorous insects. Planthoppers also tend to select nitrogen-rich over nitrogen-poor plants^15^. The combined effects of increased colonization, increased stimulation for feeding, and increased fitness generally result in rapid population growth^20^. Thus nitrogen enriched and enhanced soluble protein content host plants improve all fitness traits of BPH since the fitness traits of BPH followed the same direction as the N factor (Fig. 1).

Phosphorus enriched plants showed similar effect on the several fitness traits of BPH. Phosphorus is considered as an important component for the growth of phytophagous insects because it helps in RNA synthesis, when phosphorous is limiting, severe consequences on the fecundity, growth rate, body size, oviposition and survivorship of plant herbivores can occur^44–50^. Potassium was found to have no significant effect in this experiment, but K has a critical role in plant physiology^51^, affecting for example the distribution of primary metabolites in plant tissues, which in turn could affect the attractiveness of the plant for insects as well as their subsequent growth and development on the plant. However, other research has found that a higher rate of potassium fertilizer was associated with lower BPH population^52^. This might be due to decrease in soluble protein and free sugar content in potassium fertilized plants. In this factorial fertilizer study, we found that soluble proteins and free sugars were significantly reduced by higher levels of potassium fertilized rice plants, which strongly correlated with BPH population numbers and their dry weight. A deficiency of potassium in rice plants has been found to increase population buildup of white-backed planthopper, *Sogatella furcifera* (Horváth) while application of a high dose of potassium to rice plants decreased population buildup of this insect^52–54^. Furthermore, our experiment showed that higher nitrogen fertilizer input decreased Si content in host plant tissue, since they are negatively correlated (Fig. 1). Numerous studies have shown Si applications can enhance plant resistance to insect herbivores and other arthropods^55–57^. Surprisingly, we find that elevated nitrogen content of plant tissue contains less amount of silicon (Fig. 6), which suggests an interactive N - Si mechanism that can highly favour increases (or decreases) in BPH infestation in rice fields. Indeed, nitrogen is considered as a limiting nutrient factor for the growth of many herbivores because immature stages may experience lack of organic nitrogen (*i*.*e*., specific protein and/or amino acids) leading to reduced or impaired metabolism during portions of their critical growth period, leading even to premature death^58–60^.

High Si plants are less palatable/susceptible to insects, and BPH suffered a higher mortality rate during the nymphal stage when reared on nitrogen-limited host plant. In addition, lower host nutritional value may influence the feeding behaviour of the immature stage of insect^43^. We found BPH had a longer development time (*i*.*e*., delayed development). One possible explanation could be that nymphs must compensate for the insufficient nitrogen-based food by increasing daily food intake or switching foraging activity toward nitrogen-richer plants (if possible) or plant organs^61–63^. In this study, BPH nymphs were confined in a mesh cages over potted rice plants, restricting them so they could not forage in other areas having rice plants richer in nitrogen. In addition, nymphs were limited to feed only on the bases of the rice plant and not leaves or other parts. Therefore, the nymphal development might be delayed due to lower food quality and/or quantity (e.g., lower nitrogen-based nutrients).

Nitrogen-limited rice plants could have higher levels of chemical defenses against BPH. The Growth Differentiation Balance Hypothesis (GDBH) predicts moderate growth limitations caused by external factors such as low nutrient availability result in an accumulation of carbohydrates and, subsequently, in increased concentrations of constitutive secondary compounds^64^. Indeed, we assumed that the nitrogen-limited rice plants produced more insect-defensive soluble phenolics (C-based defensive compounds such as chlorogenic acid, rutin, and kaempferol-rutinoside)^64–68^, and along with an increased concentration of constitutive Si, a key defensive compound in rice plant, found in this study to negatively affect BPH fitness traits, as nitrogen content decreased (Fig. 1). Alternatively, for lessened N, silicon acts as a defensive chemical against insect herbivores, matched with lowered accumulation of soluble protein. Therefore, we also propose that plants excessively supplied with nitrogen have reduced insect-defensive soluble phenolics^64–69^.

Too much nitrogen, on the other hand, applied to rice plants triggered favourable fitness traits of BPH. Our research report for the first time that excessive nitrogen inputs increases *r*_*m*_ of a Hemipteran herbivore while significantly decreases the generation time by doubling the population time. In addition, high nitrogen fertilization decreases plant Si content and soluble proteins and thereby appears to reduce insect-defensive mechanisms.

Our research results indicate that K has no significant effect of BPH fitness traits. However, Liu *et al*.^70^ reported that low (20 mg/L) and highest K levels (160 mg/L) in hydroponic solution lowered K level in plants than the control, which facilitated the fecundity of BPH. These results slightly differed from our results. It can be explained that variation may be occurred due to different crop growing media. Liu *et al*.^70^ used hydroponic system which supplied only the added elements to the plant. Therefore, other defensive chemical were absent in the system but we used soil which can supply various element to plant that could affect the effect of K. Besides this, our previous report indicated that the concentration of K in rice plant is higher than the body K content of BPH^34^, suggesting that K is not limiting element for BPH. Therefore, we can assume that K has no significant effect of BPH fitness traits.

The findings of this study suggest that the rice plant itself is involved with a possibly novel N by P by K by Si by BPH interaction, and to see why, consider some past citations. An assessment of the nitrogen effect on rice soils in Bengal was reported nearly 80 years ago, when De^71^ stated that the persistence of rice grown in the same field year after year without depleting soil fertility is ‘well known’ (emphasis added). He provides results from some experiments to assess whether rice can fix nitrogen from the air, or obtains its nitrogen supply from microorganisms in the soil, of which these instead provide the supply. He concludes from the results that N may be supplied to rice in part by both mechanisms. Another report several decades later, examines nutritional disorders of rice in Asia^72^, and defines these as ‘any abnormality of rice caused by a deficiency of any essential element, any toxicity caused by a high level of any substance or ion in the soil, and any retarded growth due to a high osmotic pressure of the soil solution’. The reports states that a lack of N causes stunting and limits the number of tillers. And, leaves are narrower, shorter, and yellowed, except for young leaves which are greener, while older leaves die when becoming a light straw color. Lack of P also causes stunting, narrow and short leaves of a dirty dark green color and limits the number of tillers. Young leaves are healthier than older leaves, and old leaves die when becoming brown colored. Symptoms differ if the rice is a variety that is able to produce anthocyanin pigments. Limited supply of K also stunts plants but does not usually reduce tiller number. Leaves are short, droopy and dark green, where lower leaves exhibit yellowing at the inter-veins, starting first at the tip, and which eventually turn to a light brown color. At times, brown spots may develop on dark green leaves. The report further discusses deficiency symptoms for S, Ca, Mg, Fe, Mn, Z, B, and Cu, and for low Si content, they remark that leaves become soft and droopy. Toxicity symptoms arise with high Fe, Mn, B, Al, and also with salt injury. A table of ranges for deficiency and toxicity of these elements is also provided. From that table, N deficiency is reported as at 2.5% from leaf assay during tillering, P deficiency is at 0.1% from leaf assay at tillering, and a P toxicity is at 1% by assay of straw at maturity, and for K, a deficiency is at 1% by leaf assay during tillering. Comparison of these values with the tabulated values of the report, and plots of our experiment, indicates most of our results are near some of these reported ranges for N, P and K when rice shows symptoms.

Our findings support the “Plant vigor hypothesis”, as BPH survived better and developed faster on the rice plants with better growth status (e.g., a larger plant growth rate in terms of height, biomass and tiller numbers (Fig. 1)). Higher nitrogen and phosphorus fertilizer inputs improved all fitness traits of BPH resulting significant higher number of F_1_ population from one pair of adult^46, 47, 50, 72–78^. BPH required more time to develop in order to compensate for the nitrogen deficiency and increased Si; however, despite a longer development time, optimal body weight was not reached.

## Conclusion

Results from this study support some causal reasons for the observed and wide spread outbreaks of BPH in rice fields across Asia. Field outbreaks of BPH are also caused by wide spread use of chemical insecticides. Broad spectrum insecticides negatively impacts on BPH natural enemies that could induce huge population in field^79^. However our results also support that higher fertilizer could trigger BPH by reducing population developmental time and defense chemicals in host. So the manipulation of fertilizer inputs, especially of nitrogen regimes, may help to optimize agricultural practices by promoting negative effects on herbivorous insects. This could actually be achieved with little damage to plants and negligible crop yield^80–82^. Future work should aim at quantifying the trade-off between the negative impact on herbivorous pests and plant yield using sub-optimal, optimal, excessive and limited fertilizer input, as well as including effects of fertilizers on arthropods from the higher trophic level (i.e. natural enemies). In addition experiments are needed also to investigate to what extent fertilizer regimes may affect biocontrol agents and the performance of insecticide applications, with both variables being tested independently and in combination. Finally, field experiments are needed to confirm the data obtained from experimental greenhouse studies. Without including the effects of fertilizers on insect fitness parameters, the design of more innovative technologies that are mostly self-operating with minimal inputs^83^ for the control of crop pests is difficult.

## Materials and Methods

### Experimental units

Individual pots (16 cm height and 14 cm diameter) with two kg of soil and six rice plants (three hills with two seedlings in each hill) were used as experimental units. Some assayed soil characteristics were a pH 5.3, a 0.72% (of dry weight) organic C, as well as being deficient in nitrogen, phosphorous, and potassium. All combinations of the following fertilizer^27^ regimes were established by applying: 1) 0 (denoted “N_0_”), 100 (denoted “N_100_”), or 200 (denoted “N_200_”) kg/ha of additional nitrogen (urea, applied as CH_4_N_2_O) ha^−1^, 2) 0 (denoted “P_0_”), 20 (denoted “P_20_”), or 40 (denoted “P_40_”) kg of additional phosphorous (triple super phosphate, P_2_O_5_) ha^−1^, and 3) 0 (denoted “K_0_”), 60 (denoted “K_60_”), or 120 (denoted “K_120_”) kg of additional potassium (K_2_O) ha^−1^. Half of the nitrogen fertilizer amount but the whole of phosphorous and potassium were mixed into the soil prior to planting. The remaining half of the nitrogen amount was applied when rice plants were 30-day-old (primary tillering stage). Fifteen-day-old rice seedlings of variety BR3 were transplanted into each pot, and water was supplied when necessary. These chosen amounts of fertilizer regimes and the selected rice variety conform to expected practices under Bangladesh production scenarios. The potted plants were grown in net house at the Bangladesh Rice Research Institute (BRRI), Gazipur, Bangladesh. Each experiment was replicated four (4) times.

### Rearing of brown planthopper (BPH)

BPH individuals required for the experiments were obtained from a mass culture maintained in a net house throughout the period of study with the susceptible variety BR3 as described by Ali *et al*.^84^. Briefly, 45–60 days old potted rice plants were cleaned and the outer leaf sheaths were removed to obtain non-infested rice plants. Potted plants were kept in an iron framed rearing cage covered by a fine mesh wire net on a tray filled with water to one-third of its height, to keep the soil moist. Gravid BPH adults were released in a cage with the rice plants for oviposition and removed 24 hours later with the help of an aspirator. Potted plants with BPH eggs were shifted every day to a series of rearing cages for hatching of eggs. After hatching, fresh potted plants matching the appropriate factorial level and phenological maturity were assigned to a cage and replaced at 3–4 days intervals to provide sufficient, fresh food resources for the developing BPH populations.

### Nutritional composition of rice plants

Effects of fertilizer regimes on the nutritional composition of rice plants were determined from four randomly chosen experimental units per treatment combination. Selected rice plants were harvested at mid-tillering stage and oven dried at 70 °C ± 5 °C for 72 h. Plant samples were ground in a Wiley Mill and analyzed for nitrogen, phosphorus, potassium, and soluble proteins (SP) as percentage of dry weight from individual plants. Nitrogen was determined volumetrically by micro Kjeldahl distillation method as described by Yoshida *et al*.^85^. For determination of phosphorus and silicon, 1 g samples from each treatment were digested with a 10-ml of acid mixture of HClO_4_, HNO_3_ and H_2_SO_4_ (300: 750: 150-ml) on a hot plate at 500 °C until a gelatinous white residue remained to which distilled water was added after cooling. The mixture was filtered through Whatman #42 filter paper. Thereafter, phosphorous was determined calorimetrically through a spectrophotometer at a 420 nm wave length^86^. The filter paper and residue of the sample extract was dried in an oven at 80 °C. Then the residue on filter paper was burnt at 500 °C for 4 to 5 h. The ash was cooled in a desiccator for at least 2 h before weighing. This gravimetric determination gave an estimate of crude silica in 1 g of the dry plant sample^86^. For analysis of potassium, 1 g of plant sample was soaked for 24 h in 25-ml 1 N HCl and filtered through Whatman #42 filter paper. Potassium was analyzed in the filtrate using a Flame Photometer at a 214 nm wave length^86^.

Total free sugar content was estimated by the Anthrone method described by Yoshida *et al*.^85^. A ground sample (100 mg) was placed into a 15-ml centrifuge tube to which was added 10-ml of 80 percent ethanol. A glass ball on top of the tube was placed and kept in a water bath at 80° to 85 °C for 30 mins and then centrifuged and decanted into a 50-ml beaker. The alcohol extract was evaporated on a water bath at 80–85 °C to remove most of the alcohol. Sugar extract (5-ml) was transferred to a 100-ml volumetric flask and made up to this volume with distilled water. Five-ml of this diluted sugar extract was put into a Pyrex test tube and then sample tubes and the tubes containing the standards were placed into an ice bath. To each tube, 10-ml of the anthrone reagent were slowly added allowing the reagent to run down the side of the test tube, which were put into a boiling water bath for 7.5 min and then cooled immediately in ice. When cooled, the absorbance was measured at 630 mμ^86^.

Soluble protein content was estimated according to Lowrys method^85^. Different dilutions (0.05 to 1 mg/ml) of bovine serum albumin (BSA) solutions were prepared by mixing stock BSA solution (1 mg/ml) and water in the test tube. The final volume in each of the test tubes was 5-ml. From these different dilutions, 0.2-ml protein solution was pipetted to different test tubes and 2-ml of alkaline copper sulphate reagent (analytical reagent) was added and the solutions mixed. This solution was incubated at room temperature for 10 min. Then 0.2-ml of Folin reagent solutions were added to each tube and incubated for 30 min and the absorbance at 660 nm was measured. The absorbance against protein concentration was plotted to get a standard calibration curve. The absorbance of each unknown sample assayed and the concentration was determined using the standard curve plotted above^86^.

### Fitness traits of BPH

BPH nymphal survival, duration of instars, body weight of adult females, adult longevity, fecundity (r_m_), egg hatchability and number of individuals successfully developed to adults (R_0_) from a single pair of insects are the estimated fitness traits^20^ in this study.

#### Nymph survival and development

Additional experimental units (or single pots) from the necessary factorial combinations, that consisted of 40 day-old (at the maximum tillering stage) fresh plants were trimmed back to six tillers and placed in mylar-cages (made from transparent thick plastic paper, BRRI). Twenty newly hatched first instar BPH nymphs were then introduced into each mylar-cage. Observations of surviving nymphs and emerging adults were conducted on a daily basis after release. The number of nymphs that reached adult stage and the time required to complete the nymphal period were recorded. Fresh weight of adults was also measured.

#### Adult longevity, fecundity and egg hatchability

Five pairs of newly molted adults from BPH cultures reared under different fertilizer regimes were introduced into other mylar-cages with 45 day-old fresh BR3 rice plants (maximum tillering stage, trimmed to 6 tillers in each cage) also from appropriate fertilizer factorial combinations. The numbers of surviving adults were recorded daily until their death. Newly hatched nymphs were counted and removed. After all adults had died, destructive sampling was conducted to determine the number of unhatched eggs. Stems were cut at the base and kept in test tubes containing 70% ethanol to dissolve the chlorophyll for easy detection of eggs. Subsequently, individual stems were dissected under a binocular microscope for counting the number of unhatched eggs and to calculate egg hatchability. Lifetime fecundity was calculated as the summed number of nymphs counted and total number of unhatched eggs found per caged BPH female after her death.

#### BPH produced and dry weight gained

Individual pairs of newly molted BPH adults were transferred to another experimental unit of the appropriate fertilizer factorial combination and then maintained inside other individual, transparent, mylar-cages to quantify population growth for a period of 25 d. Males and females were checked after 24 h of infestation, and dead males or females were replaced with newly molted BPH adults if any had died within the initial 24 hours after being caged. At 25 d after release of the adults, the first generation (F_1_) BPH individuals were collected, transferred to ethanol (70%) and counted. BPH individuals were dried in the oven at 60 °C until constant weight at 48 h and the dry weight of BPH was measured.

### Modeling dynamics of BPH populations

Daily age-specific survival (*l*_*x*_) and fecundity (*m*_*x*_) rates were used to generate life tables for each fertilizer regime. The intrinsic rate of natural increase (*r*_m_) was estimated from the life-fecundity table according to the equation 2 ^87^:

2 $$[\sum {e}^{-rmx}{l}_{x}{m}_{x}=1],$$

in which *x* is female age in days, *l*_x_ is the age-specific survival rate [(fraction of females surviving at age *x*) × (rate of egg hatchability) × (survival rate of immature stages)] and *m*_*x*_ is the expected number of daughters produced per female alive at age *x* [(age-specific oviposition) × (proportion of females)]. The net reproductive rate (*R*_0_) is given by equation 3:

3 $$[{R}_{0}=\sum {l}_{x}{m}_{x}],$$

the mean generation time (*t*_G_) in days is given by equation 4:

4 $$[{t}_{G}=\,\mathrm{ln}\,{R}_{0}/{r}_{m}],$$

the finite rate of increase (*λ*) is given by equation 5:

5 $$[\lambda ={e}^{rm}],$$

and the doubling time (*t*_D_) in days is equation 6:

6 $$[{t}_{D}=(\mathrm{ln}\,2)/{r}_{m}].$$

After *r*_m_ was computed from the original data (*r*_all_), the standard errors for life-history parameters at different constant fertilizer inputs were estimated using the Jackknife method^88, 89^. Briefly, one BPH individual is omitted at a time and *r*_m_ (*r*_*i*_) is calculated for the remaining BPH (*n*-1). Based on Meyer *et al*.^88^, the Jackknife pseudo-value (*r*_*j*_) is computed for this subset of the original data according to the equation 7:

7 $$[{r}_{j}=n\,{r}_{{\rm{all}}}-(n-1){r}_{{\rm{i}}}]$$

This process was repeated for all possible omissions of one BPH individual at a time from the original dataset to produce pseudo-values which allowed for computing confidence limits for the parameter values.

### Statistical analyses

Data were analyzed by means of analyses of variance (one-way ANOVA) in SPSS version 16^90^, using fertilizer inputs as the explanatory variable. Response variables were subjected to a logarithmic or arcsine transformation prior to analysis to stabilize the variance, and it was confirmed that the back-transformed values were non-negative. The mean differences among treatments were compared by Tukey’s honest significance test with SPSS software. First, principal component analysis (PROC PRINCOMP) in SAS^41^ was used to examine associations/disassociations among: fertilizer regimes, nutritional elements (nitrogen, phosphorous, potassium, silicon, total free sugar, and soluble protein) of rice plants, and BPH fitness traits.

Second, PROC GLMSELECT in SAS (Enterprise Guide 6.1) identified the model that best fit the intrinsic rate of natural increase (*r*_*m*_) and reproduction rate (*R*_0_). The full model for *r*_*m*_ was given equation 8:

8 $$\begin{array}{rcl}{r}_{m} & = & {\beta }_{0}+{\beta }_{1}K+{\beta }_{2}P+{\beta }_{3}N+{\beta }_{4}{K}^{2}+{\beta }_{5}{P}^{2}\\  &  & +\,{\beta }_{6}{N}^{2}+{\beta }_{7}KP+{\beta }_{8}KN+{\beta }_{9}PN+{\beta }_{10}KPN+\varepsilon \end{array}$$

The full model for *R*_0_ was given by equation 9:

9 $$\begin{array}{rcl}{\rm{In}}\,{R}_{0} & = & {\beta }_{0}+{\beta }_{1}K+{\beta }_{2}P+{\beta }_{3}N+{\beta }_{4}{K}^{2}+{\beta }_{5}{P}^{2}\\  &  & +\,{\beta }_{6}{N}^{2}+{\beta }_{7}KP+{\beta }_{8}KN+{\beta }_{9}PN+{\beta }_{10}KPN+\varepsilon \end{array}$$

Third, statistical analyses of relationships among fertilizer regimes, nutritional elements (nitrogen, phosphorous, potassium, silicon, and soluble protein) of rice plants, and the BPH fitness traits was done by MANOVA using PROC GLM in SAS software (SAS Institute, Cary, NC, USA)^91^. With the MANOVA, 27 treatment combinations were coded into a new variable (TRT) and two other MANOVAs for nutritional elements and fitness traits were performed without data transformations, where in each, the levels of the N factor were used as a BY variable. If the overall MANOVA was significant, an interpretation of the univariate ANOVAs and Tukey tests were obtained^92^. The treatment combination effects of the MANOVA for the fertilizers can, for instance, be stated as the following multivariate null hypothesis:

H_0_: In this experimental population, there are no differences between treatment combinations involving N, P and K within the three different levels N_0_, N_100_, or N_200_ when assayed physiological characteristics of rice plants are compared simultaneously.

This null hypothesis can be symbolically represented by equation 10:

10 $$(\begin{array}{c}\begin{array}{c}{M}_{11}\\ {M}_{21}\end{array}\\ {M}_{31}\\ {M}_{41}\\ {M}_{51}\end{array})=(\begin{array}{c}\begin{array}{c}{M}_{12}\\ {M}_{22}\end{array}\\ {M}_{32}\\ {M}_{42}\\ {M}_{52}\end{array})=(\begin{array}{c}\begin{array}{c}{M}_{13}\\ {M}_{23}\end{array}\\ {M}_{33}\\ {M}_{43}\\ {M}_{53}\end{array})=\cdots =(\begin{array}{c}\begin{array}{c}{M}_{17}\\ {M}_{27}\end{array}\\ {M}_{37}\\ {M}_{47}\\ {M}_{57}\end{array})=(\begin{array}{c}\begin{array}{c}{M}_{18}\\ {M}_{28}\end{array}\\ {M}_{38}\\ {M}_{48}\\ {M}_{58}\end{array})=(\begin{array}{c}\begin{array}{c}{M}_{19}\\ {M}_{29}\end{array}\\ {M}_{39}\\ {M}_{49}\\ {M}_{59}\end{array})$$

where, for *k* = 0, 100, 200, let element *M*_*ij*_ represent a population mean for the treatment factorial combinations of the factors N, P, and K recoded as a new variable (TRT_*l*_, or *l* = 1, 2, 3, …, 25, 26, 27). The first number in the subscript pair (*ij*) identifies one the physiological characteristics in list order (*i* = nitrogen, phosphorous, potassium, soluble protein and silicon) of assayed rice plants. The second number of the subscript pair identifies which one of the nine possible treatment groups found at each level of the nitrogen factor. Thus, for the variable TRT_*l*_, equation 9: *l* = (*j* + *k*) = 1, 2, 3, …, 9; or 10, 12, 13, …, 18; or 19, 20, 21, …, 27, for N_0_, N_100_, or N_200_, respectively.

Results from PCA and MANOVA led to further analyses with PROC GLIMMIX and SAS^®^^91^ data step programming to produce two stylized, multi-dimensional plots of selected variables of the diverse types (e.g., factors, plant nutritional elements and BPH fitness traits) to visualize subtleties reported in this paper for consideration in the design of future experiments in either the net house or field plots. Several concepts (i.e., hexadecimal RGB (Red, Green, Blue) color codes) and software packages (SAS/GRAPH software (SAS Institute, Cary, NC, USA) and ArcGIS software by ESRI (Redlands, CA, USA)) combined the various results of this study to generate these stylized multi-dimensional plots^93^.
